# Potential Cost-Effectiveness of Prenatal Distribution of Misoprostol for Prevention of Postpartum Hemorrhage in Uganda

**DOI:** 10.1371/journal.pone.0142550

**Published:** 2015-11-11

**Authors:** Solomon J. Lubinga, Esther C. Atukunda, George Wasswa-Ssalongo, Joseph B. Babigumira

**Affiliations:** 1 Pharmaceutical Outcomes Research and Policy Program, Department of Pharmacy, University of Washington, Seattle, Washington, United States of America; 2 Global Medicines Program, Department of Global Health, University of Washington, Seattle, Washington, United States of America; 3 Department of Pharmacology, Mbarara University of Science and Technology, Mbarara, Uganda; 4 Department of Obstetrics and Gynecology, Mbarara Regional Referral Hospital, Mbarara, Uganda; Mahidol-Oxford Tropical Medicine Research Unit, THAILAND

## Abstract

**Background:**

In settings where home birth rates are high, prenatal distribution of misoprostol has been advocated as a strategy to increase access to uterotonics during the third stage of labor to prevent postpartum hemorrhage (PPH). Our objective was to project the potential cost-effectiveness of this strategy in Uganda from both governmental (the relevant payer) and modified societal perspectives.

**Methods and Findings:**

To compare prenatal misoprostol distribution to status quo (no misoprostol distribution), we developed a decision analytic model that tracked the delivery pathways of a cohort of pregnant women from the prenatal period, labor to delivery without complications or delivery with PPH, and successful treatment or death. Delivery pathway parameters were derived from the Uganda Demographic and Health Survey. Incidence of PPH, treatment efficacy, adverse event and case fatality rates, access to misoprostol, and health resource use and cost data were obtained from published literature and supplemented with expert opinion where necessary. We computed the expected incidence of PPH, mortality, disability adjusted life years (DALYs), costs and incremental cost effectiveness ratios (ICERs). We conducted univariate and probabilistic sensitivity analyses to examine robustness of our results. In the base-case analysis, misoprostol distribution lowered the expected incidence of PPH by 1.0% (95% credibility interval (CrI): 0.55%, 1.95%), mortality by 0.08% (95% CrI: 0.04%, 0.13%) and DALYs by 0.02 (95% CrI: 0.01, 0.03). Mean costs were higher with prenatal misoprostol distribution from governmental by US$3.3 (95% CrI: 2.1, 4.2) and modified societal (by US$1.3; 95% CrI: -1.6, 2.8) perspectives. ICERs were US$191 (95% CrI: 82, 443) per DALY averted from a governmental perspective, and US$73 (95% CI: -86, 256) per DALY averted from a modified societal perspective.

**Conclusions:**

Prenatal distribution of misoprostol is potentially cost-effective in Uganda and should be considered for national-level scale up for prevention of PPH.

## Introduction

Postpartum hemorrhage (PPH) is the most important contributor to maternal burden of disease in sub-Saharan Africa. It is estimated to occur in up to 10.8% of pregnancies [1], is the leading contributor to disability adjusted life years (DALYs) among pregnancy-related complications [2] and accounts for 15.2% of maternal deaths in sub-Saharan Africa [3]. In resource-limited settings, access to oxytocin (10 international units administered intramuscularly), the first line uterotonic for prevention of PPH is limited because: 1) it can only be administered by skilled birth attendants [4]; 2) health facility birth rates are low—57.4% of births take place in a health facility with skilled supervision (i.e. a doctor or nurse/midwife); 42.6% of births take place outside health facilities (18.3% are assisted by traditional birth attendants, 15.3% by relatives/friends, 1.9% by clinical officers/medical assistants and 7% are unassisted) [5]; 3) health centers lack cold chain storage necessary to maintain long-term stability of oxytocin [6], and 4) it is regularly stocked-out due to poor forecasting and supply management [6].

Misoprostol (600μg) could increase the number of births in resource-limited countries that are covered by a uterotonic [7,8]. There are variations in the effectiveness of misoprostol by delivery practices. Although the effect of misoprostol on PPH in the presence of skilled birth attendance (where the active management of third stage of labor (AMTSL) is practiced) is less marked [9], it offers several advantages: 1) it is more effective than placebo in unskilled and home births [10,11], 2) it is inexpensive, 3) it can be transported and stored without refrigeration, and 4) it can be administered without an injection and could be used by unskilled birth attendants or by the mother herself. Previous cost-effectiveness analyses of misoprostol for the prevention of PPH have focused on home births [12,13], or births attended by traditional birth attendants (TBAs) [14]. However, there are no good published models to predict which women are likely to deliver at home or with TBAs.

Community misoprostol distribution has been advocated as a strategy to increase access to a uterotonic immediately following delivery of the baby [15,16]. Several strategies for distribution have been suggested, including through frontline health workers (trained TBAs, auxiliary midwives and community health workers), as well as distribution directly to women at a prenatal visit [17,18]. In this analysis, we examine the potential cost-effectiveness of prenatal community distribution of misoprostol to pregnant mothers as a strategy to increase access to uterotonics for prevention of PPH in Uganda. The goal of the strategy is not for misoprostol to replace oxytocin as a first line uterotonic; rather, that women who do not receive oxytocin immediately following delivery, either because they are unable to access a health facility, or because the facility cannot provide parenteral oxytocin, would face an increased probability of receiving misoprostol as a second line uterotonic [19].

## Methods

### Modeling framework

**Fig 1:**
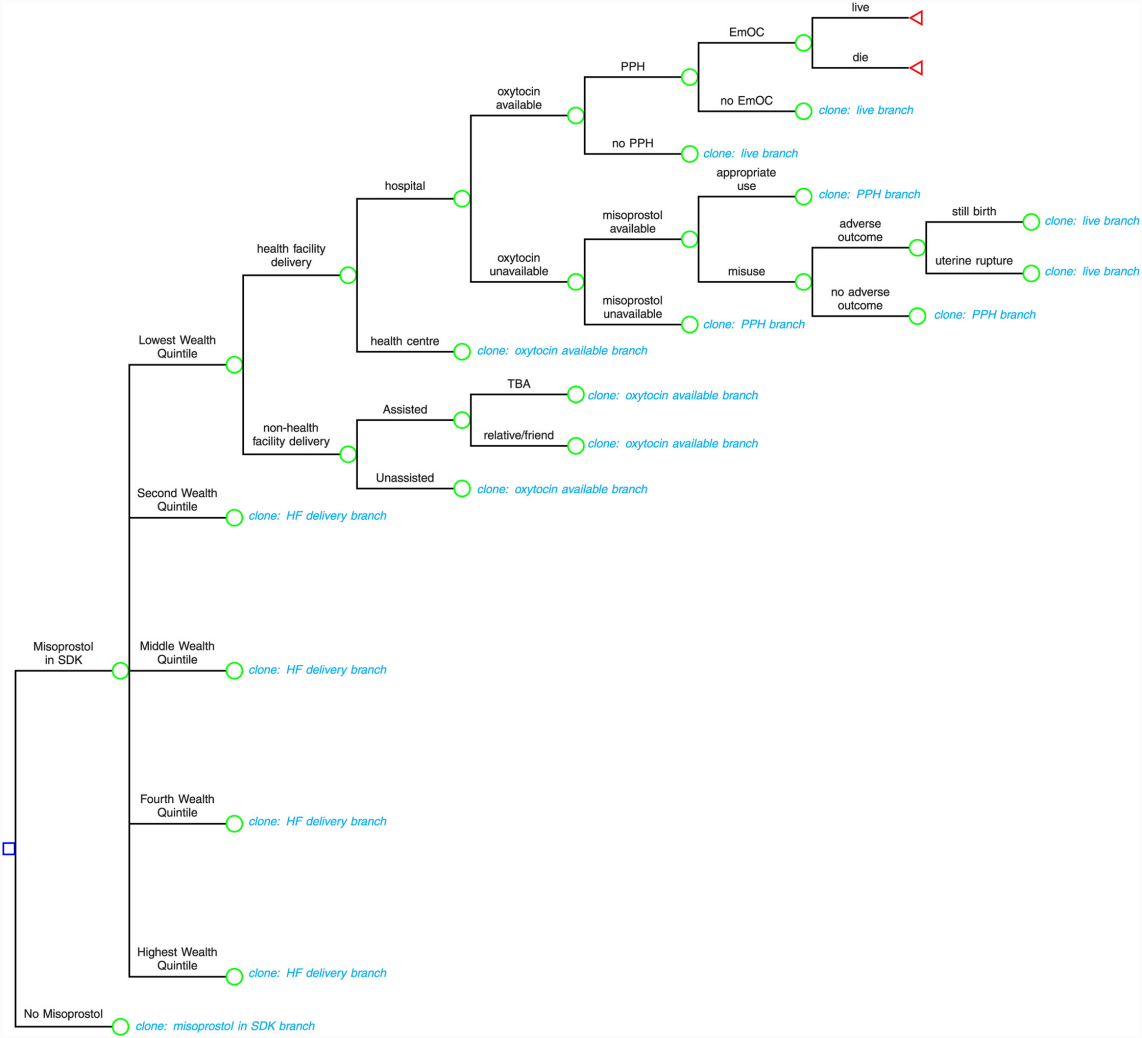
Decision tree showing the delivery pathway trajectory and outcomes considered in the analysis. The delivery pathway trajectory i.e., place of delivery (health facility versus non-health facility birth) and assistance at delivery (skilled assistance, traditional birth attendant, relative or friend and unassisted delivery) is defined by wealth quintile from the Uganda Demographic and Health Survey. We follow women through to the immediate postpartum period in which they may receive prophylactic uterotonics (or not), may experience postpartum hemorrhage (or not), may access emergency obstetric care or not and may recover or die due to postpartum hemorrhage. We allow access to emergency obstetric care to vary by delivery pathway. Further, we account for the potential of misoprostol misuse through stillbirth or uterine rupture outcomes. This model structure is used to project the costs and outcomes (incident postpartum hemorrhage, mortality due to postpartum hemorrhage and disability adjusted life years) of a cohort of pregnant women in Uganda.

A decision model was developed in Microsoft Excel (2011) to compare two strategies: 1) misoprostol (600μg taken orally immediately following delivery of the baby) distributed to all mothers either at a prenatal visit in the third trimester or as an add-on to a safe delivery kit (SDK) and 2) only oxytocin (10 international units administered intramuscularly immediately following delivery of the baby), whose use is limited to births in health facilities, is available (Fig 1). Prenatal visits are ideal for distribution because over 90% of mothers will visit a skilled provider at least once [5]; and addition to a safe delivery kit is potentially efficient because it would leverage an already existent service, and could ensure that mothers are aware that the drug should be used as part of the delivery procedure.

We tracked the potential delivery pathways and outcomes of a mother in Uganda (Fig 1) from the prenatal period, through labor and delivery, to the immediate postpartum period in which she could experience PPH and either survive or die due to PPH. Data from the Uganda Demographic and Health Survey (UDHS) 2011 [5] show that 47.6% of births take place outside health facilities, without skilled attendance. Some commonly cited reasons for delivery outside health facilities include the inaccessibility of level III health facilities (the lowest level that should offer maternity services) either due to distance required to travel or lack of transportation and money to deliver in facilities [5,20]. Other reasons for delivery outside health facilities include: the sudden onset of labor and short labor which may preclude making it to the health facility; facility-based factors such as poor staff attitudes and lack of privacy; and sociocultural factors like the lack of power in decision-making in relation to delivery [21].

Maternal age, education, child birth order, region, rural or urban residence and economic status are important determinants of maternal delivery pathway trajectories—i.e., both place of delivery (health facility versus non health facility) and the type of assistance at delivery (skilled versus unskilled assistance). Younger (less than 20 years), more educated (with at least a secondary education) women, first order births, women from Kampala, those who resided in urban areas and women in the highest economic quintile were more likely to deliver in health facilities with skilled attendance [5,20]. The wealth index in the UDHS is a useful summary proxy of key variables (maternal education, region, urban versus rural residence and economic status), that drive maternal delivery trajectories [5,20]. Therefore depending on the UDHS wealth index [5], a mother could deliver either at a hospital or health center (with skilled assistance) or out of the health facility (either assisted by TBAs, friend or relative, or without assistance). Additionally, structuring the model according to wealth index provides a useful way to demonstrate the distribution (heterogeneity) of outcomes across wealth strata in a population [22].

The model allowed for differential access to uterotonics and emergency obstetric care (EmOC) by delivery pathway trajectory [23,24]. A mother could receive the first line drug, oxytocin if she delivered at a health facility (hospital or health center). If oxytocin was unavailable at the health facility or if she delivered outside a health facility (assisted by a TBA, relative/friend or by herself), she could receive either misoprostol or no uterotonic. If she experienced PPH, a mother may access EmOC at a hospital or not, depending on where she delivered. Finally she may die or survive. There are several published case reports of stillbirths and vaginal rupture following the misuse of misoprostol prior to birth of the baby [25–30]. We therefore accounted for this possibility in our model.

### Outcomes

The outcomes in the model were PPH, death due to PPH, DALYs and costs associated with delivery, PPH prevention and treatment. We estimated costs from the governmental (the relevant payer in Uganda) and modified societal perspectives [31]. The governmental perspective considered direct medical costs (including health worker time, drugs, sundries, and laboratory tests) and some direct non-medical costs (health facility overhead and capital costs). The modified societal perspective considered additional direct non-medical costs (out-of-pocket travel and upkeep costs), and indirect costs (time costs for the mother and her care giver). Future outcomes were discounted at 3% [32].

### Model assumptions

1. While misoprostol, distributed this way, could be misused to induce abortion, demonstration projects in Tanzania, Ghana, Nigeria and Uganda found this highly unlikely as close to 100% of women demonstrated appropriate use (within a minute of delivery) [33]. There were no reported cases of diversion of misoprostol to induce abortion.
2. We assumed, in our base case, that distribution of misoprostol did not alter the likelihood of health facility delivery. The reality is that the likelihood of health facility delivery could either increase (as a consequence of additional prenatal counselling and confidence in the health service) or decrease (as misoprostol distribution could incentivize home delivery) [6,15].
3. We assumed, for simplicity that all women in the model underwent vaginal deliveries.

### Delivery pathway trajectories of a mother

Data from UDHS, 2011 were used to estimate the probabilities for each delivery pathway trajectory of a mother in Uganda by wealth quintile (Table 1) [5].

**Table 1:** Parameters to compute a woman’s probable delivery pathway trajectory: base case probabilities (sensitivity ranges) by wealth quintile, UDHS 2011[5].

Parameter	Lowest	Second	Middle	Fourth	Highest	Distribution
Proportion in quintile	0.224 (0.215, 0.234)	0.214 (0.205, 0.223)	0.200 (0.191, 0.209)	0.177 (0.168, 0.185)	0.185 (0.177, 0.194)	Dirichlet
Probability of health facility delivery^1^	0.435 (0.421, 0.449)	0.489 (0.475, 0.503)	0.544 (0.530, 0.558)	0.596 (0.582, 0.610)	0.884 (0.875, 0.863)	Beta (for each)
Probability of delivery in hospital^2^	0.397 (0.377, 0.417)	0.452 (0.432, 0.472)	0.484 (0.464, 0.504)	0.531 (0.511, 0.551)	0.680 (0.669, 0.691)	Beta (for each)
Probability delivery is unassisted^3^	0.080 (0.058, 0.102)	0.095 (0.071, 0.119)	0.104 (0.079, 0.129)	0.06 (0.040, 0.080)	0.013 (0.004, 0.022)	Beta (for each)
Probability delivery is assisted by TBA^†^	0.232 (0.210, 0.254)	0.220 (0.197, 0.241)	0.178 (0.159, 0.198)	0.216 (0.195, 0.238)	0.056 (0.044, 0.068)	Beta (for each)

^1^ conditioned on wealth quintile

^2^ conditioned on delivery in health facility

^3^ Joint probabilities of non-health facility delivery and either unassisted or assisted by TBA

^†^TBA = Traditional Birth Attendant

### Access to uterotonics and emergency obstetric care

Access to uterotonics was defined as the probability that a mother in the third stage of labor would receive a uterotonic. Data from two health facility based studies were used to estimate probabilities of receipt of oxytocin [23,24] and data from a demonstration project in Tanzania, an East African country similar to Uganda culturally and in maternal health seeking behaviors, were used to estimate probabilities of receipt of misoprostol (Table 2) [33]. The third author, a consultant obstetrician and gynecologist in Uganda provided expert professional opinion on access to EmOC for women who develop PPH. We define access to EmOC as access to a facility that provides the basic signal functions: parenteral antibiotics, and oxytocics, manual removal of placenta, removal of retained products and assisted vaginal delivery [23,24].

**Table 2:** Probabilities of receiving uterotonics and probabilities of accessing emergency obstetric care by delivery pathway trajectory.

Parameter	Base case	Sensitivity Range	Distribution	Source
Probability of receiving oxytocin				
Hospital	0.887	0.842, 0.931	Beta	Mbonye et al [23,24]
Health center	0.744	0.699, 0.791	Beta	Mbonye et al [23,24]
Probability of receiving misoprostol				
In non-health facility births	0.911	0.879, 0.938	Beta	IHI [33]
In health facility birth (given no oxytocin)	0.744	0.696, 0.792	Beta	IHI [33]
Proportion of patients with access to EmOC				
If unassisted at delivery	0.900	0.850, 0.950	Beta	Expert opinion
If home delivery, assisted by friend/relative	0.900	0.850, 0.950	Beta	Expert opinion
If delivery assisted by TBA	0.900	0.850, 0.950	Beta	Expert opinion
If health center delivery	0.950	0.900, 1.000	Beta	Expert opinion

### Probability of PPH, treatment efficacy and case fatality rates

To obtain an estimate of the probability of PPH in unskilled births, we pooled estimates (using a random effects model, S1 Fig) of the probabilities of PPH in non-health facility births from the non-interventional arm of studies in which a uterotonic was compared to no uterotonic [10,34–36] in unskilled births (PPH probability: 0.122; 95% CI: 0.0678, 0.1763). The ancillary care, pre-labor risk assessment and practice of other components of AMTSL (other than oxytocin) may independently lower the underlying risk of PPH for women who give birth under skilled attendance in health facilities. To approximate this reduction in risk, we adjusted the above estimate and its lower and upper bounds by 63%, a ratio of the relative risk (RR) of PPH comparing active to expectant management of the third stage of labor (0.34) [37], and the RR of PPH comparing oxytocin to placebo (0.53) [38].

Oxytocin and misoprostol lower the risk of PPH; however misoprostol appears to have heterogeneous effects depending on whether it is used with or without skilled attendance at delivery (Table 3). Based on a Cochrane meta-analysis, we estimated the RR of PPH with oxytocin (versus placebo) in the setting of skilled attendance at delivery as 0.53 (95% CI: 0.38, 0.74) [38,39]. We pooled estimates from two trials (S2 Fig) [11,40], that compared the risk of PPH with misoprostol versus placebo in a setting of skilled assistance at delivery (RR 0.84; 95% CI: 0.73, 0.97); and the RR of PPH with misoprostol (versus placebo) in unskilled births was estimated from the trial of Derman et al. [10] as 0.53 (95% CI: 0.39, 0.74). These differences could be explained by differential skill level and practice of AMTSL in skilled deliveries—because the underlying risk of PPH is lower, this could make the independent effects of misoprostol less marked [41].

**Table 3:** Probabilities of PPH, treatment efficacy of uterotonics and case fatality rate of PPH.

*Parameter*	*Base case*	*Sensitivity range*	*Distribution*	*Reference*
Baseline probability of PPH in skilled delivery	7.8%	4.3%, 11.3%	Beta	Computed^a^
Baseline probability of PPH in unskilled delivery	12.2%	6.8%, 17.6%	Beta	Meta-analysis^b^
Relative risk of PPH of oxytocin in skilled delivery	0.53	0.38, 0.74	Log-normal	Westhoff et al. [38]
Relative risk of PPH of misoprostol in skilled delivery	0.84	0.73, 0.97	Log-normal	Meta-analysis^c^
Relative risk of PPH of misoprostol in unskilled delivery	0.53	0.39, 0.74	Log-normal	Derman et al. [10]
Case fatality rate from PPH for women who access EmOC	0.062	0.056, 0.068	Beta	Kaye et al. [42]
Case fatality rate from PPH for women who do not access EmOC	0.124	0.062, 0.186	Beta	Assumed based on Babigumira et al. [43]

^a^ Computed by multiplying the baseline probability of PPH in unskilled delivery by the ratio of the relative risk of PPH comparing active to expectant management of the third stage of labor

^b^ A random effects meta-analysis of incidence of PPH in the non-interventional arms of clinical studies comparing a uterotonic to no uterotonic

^c^ A random effects meta-analysis of trials that compared the risk of PPH with misoprostol versus placebo in a setting of skilled assistance at delivery

Approximately 6.2% of women who develop PPH in hospitals in Uganda die [42]. This was used as the case fatality rate (CFR) for PPH for women who access EmOC after delivering in a health facility. In general, those who do not deliver in health facilities eventually make it into care, albeit belatedly, when they experience complications. Because of the late presentation, we assumed that these had a twice-higher CFR. A similar assumption has been used in a previous study of the economic costs of induced abortions in Uganda [43].

### Misuse and potential changes in delivery pathway trajectory following misoprostol distribution

Misoprostol implementation projects and clinical trials have demonstrated very low rates of misuse of misoprostol and increases in likelihood of delivery with appropriate education. A study in Ghana found that 99% of mothers used misoprostol appropriately and the likelihood of health facility delivery increased from 30 to 69% [15]. In Uganda, the *MamaMiso* study found that 97% of women used misoprostol appropriately, and the 3% that took it after delivery show no adverse events [18]. However, there have been case reports of both still birth [25] and uterine rupture [25–30] following use of misoprostol for labor induction in women without previous cesarean deliveries. To account for this, we used the estimate of appropriate use from the *MamaMiso* study [18], and assumed *conservatively* that the 3% who took it before birth of the child experienced an adverse outcome—as there are more published case reports, we assume a 70% uterine rupture probability and 30% still birth probability. We then estimated the probability of death from uterine rupture in Uganda from Kaye et al. [42] as 0.118 and assumed that still birth was not fatal for the mother.

### Health resource use and costs

We used a microcosting approach to estimate health resources used and costs for health facility and non-health facility delivery, and for management of PPH, still births and uterine rupture at a hospital (detailed inputs in S1 Table and summary of cost model in S2 Table). Total costs in each category were obtained by multiplying health resources used by unit costs.

Estimates of health worker time, quantities of drugs and sundries and average length of hospital stay for PPH weighted by proportions of use at health centers and hospitals were obtained from the Uganda safe motherhood costing study [44]. We applied updated unit costs for drugs and sundries from local supplier price catalogue [45,46]. The unit cost of misoprostol was obtained from the Management Sciences for Health International Drug Price Indicator Guide (2012) [47]. To estimate the cost of a program of distribution of misoprostol, we further assumed that 200 health workers (to serve a cohort of 10,000 mothers) would undergo training for 5 days at US$10 per day, mother training by health workers would cost 20 nurse full time equivalents (1 FTE is the annual salary of 1 nurse for 1 year) plus an additional packaging cost (equivalent to 50% of the cost of a dose of misoprostol) (S3 Table).

Annual health worker pay was estimated from published pay schedules for health workers in Uganda [48]. The unit cost for blood transfusion was obtained from a study in Malawi, which is similar to Uganda [49]. Laboratory test costs were obtained from a hospital-based analysis in Uganda [50]. Facility and overhead unit costs were estimated from the WHO-CHOICE [51], model for estimating unit costs for hospitalizations [52], and either weighted by the proportion of patients who experience overnight stays for normal deliveries or by the average length of stay for PPH in a hospital. Average travel and upkeep costs at health centers and hospitals and the average cost for a normal vaginal delivery by a TBA (assuming no travel costs to TBA) were obtained from a costing study of maternal health services in Uganda [53]. The cost of management of still birth was assumed to be the same as that for a normal vaginal delivery because the management protocols are the same. The management protocols for uterine rupture involve either a total or subtotal hysterectomy, or repair with or without tubal ligation. As we had no data on the costs of (or proportions of women receiving) each of these surgical interventions, we assumed women underwent a *major surgical operation* equivalent to the cost of a cesarean delivery estimated from the Uganda safe motherhood costing study [44].

We computed patient and care giver time costs under each delivery pathway, for treatment of PPH and complications resulting from misoprostol misuse. For health facility delivery and management of complications, the time spent was estimated as a sum of the average travel time to the health facility [53], and the average length of stay in the health facility [44]. For TBA births, we assumed the travel time and length of stay was the same as that in a health center. For assisted home delivery, one relative or friend was assumed to stay with the mother for an average of 3 days. Lost time was valued at national GDP per capita for 2012 ($547) assuming 264 workdays in a year and 8 work-hours in a day. All costs were converted to 2012 US Dollars using the Uganda Consumer Price Indicator for health [54] and the Bank of Uganda exchange rate on December 31, 2012 [55].

### Disability adjusted life years (DALYs)

The average durations of disability due to PPH, vaginal stillbirth and uterine rupture in Uganda were assumed to be 1, 1 and 3 months respectively. The life expectancy at birth was 62.5 years [56], and the age distribution of pregnant women in Uganda was obtained from the UDHS [5]: 16.7% were less than 20 years, 69.7% were 24–34 years and 13.5% were 35–49 years old.

The principal source of disability in PPH is anemia; therefore we applied the disability weight for severe anemia (0.164, 95% CI: 0.112, 0.228) [57]. Disability weights for stillbirth and uterine rupture were equated to values for moderate (0.123, 95% CI: 0.083, 0.176) and severe abdominal pelvic problem (0.326, 95% CI: 0.219, 0.451) respectively from the 2010 Global Burden of Diseases study [57]. We estimated DALYs as a sum of years lived with disability (YLD) and years of life lost (YLL). YLD was computed as a product of the probability of experiencing a disability, disability weight and the duration of each disability. To obtain YLL, we summed the years of life lost (up to the life expectancy) and weighted this by the expected probability of death. The total DALYs were obtained by summing these weighted values. Future YLL were discounted at 3%.

### Analyses

Using this set up, we simulated a cohort of pregnant women in Uganda and computed expected costs, incidence of PPH, mortality, and DALYs for each strategy. We computed incremental costs and outcomes (changes in incidence of PPH and mortality due to PPH and DALYs averted) by subtracting the results for the status quo strategy from that of prenatal misoprostol distribution.

We performed scenario analyses to evaluate our assumptions. First, because delivery pathways depended on wealth quintile, we performed a stratified analysis to estimate incremental cost and outcomes in each wealth quintile. We conducted threshold analyses to determine the per-patient cost of misoprostol distribution, the probability of misuse and costs of collection and destruction of unused misoprostol, that would result in an ICER of US$1,641 (3 x GDP per capita) from both government and societal perspectives. Lastly, we evaluated a range of odds ratios (OR) for health facility delivery to examine the impact changes in odds of health facility delivery due to the program could have on outcomes (for simplicity and due to lack of supporting data, the OR did not depend on wealth quintile).

Univariate sensitivity analysis (USA) was conducted to determine the impact of uncertainty around parameters on incremental costs and outcomes. Parameters were assigned plausible ranges based either on 95% confidence intervals, or ±50% where assumed or based on expert opinion to represent greater uncertainty. Probabilities were capped at 1 if the upper bound exceeded this value. Unit costs for drugs and sundries were varied by ±20% because not much variation was expected as only two companies supply public sector facilities.

Probability distributions were assigned to all parameters used in the model: a dirichlet distribution for the probability of belonging to one of the wealth quintiles and the age distribution of pregnancies; beta distribution for probabilities and disability weight; lognormal distribution for relative risks and normal distribution for costs, and life expectancy at birth for women and exponential distributions for contact hours per-patient for the different health workers, length of hospital stay, duration of PPH. We performed 10,000 second order Monte Carlo simulations and calculated the 95% credible interval (95% CrI) for incremental costs and outcomes (as the values at the 2.5^th^ and 97.5^th^ percentiles). A net benefit framework was used to compute the probability that prenatal distribution of misoprostol was cost-effective. Threshold ICERs for interventions in developing countries are derived from multiples of GDP per capita [13,51,58]. An intervention is considered highly cost-effective if the ICER is less than 1 x GDP per capita and cost-effective if less than 3 x GDP per capita. Willingness to pay (WTP) per DALY was varied from 0 to $1,800. On each occasion, the net benefit for each simulation was calculated using the formula:

$$NetBenefit\;=\;ICER_{threshold}\;\times \;\Delta DALYs\;-\;\Delta Costs$$

The probability that prenatal misoprostol distribution was cost-effective was computed by determining the proportion of simulations for which the net benefit was greater than zero. This was done over the values of WTP per DALY and a Cost effectiveness acceptability curve (CEAC) was generated.

### Ethical considerations

The data used in this modelling analysis were drawn from publicly available sources, assumptions and the expert opinion of a co-author. It did not involve “human participants” as described by the University of Washington Institutional Review Board, and therefore was exempt from prior ethics approval. All authors had full access to the data and the spreadsheet model, and reviewed and approved the final version of the manuscript. The authors declare no competing interests.

## Results

### Cost-effectiveness analysis

In the base-case, the expected incidence of PPH was lower with prenatal misoprostol distribution (4.5% versus 5.5%; an absolute reduction of 1.0% and relative reduction of 23.8%). The probability of death due to PPH was lower with prenatal misoprostol distribution (0.28% versus 0.36%; an absolute reduction of 0.08% and relative reduction of 27.2%). Mean DALYs were lower with prenatal misoprostol distribution (0.06 versus 0.08; equivalent to 0.02 DALYs averted). Mean costs were higher with prenatal misoprostol distribution from both the government ($15.6 versus $12.3, an incremental cost of $3.3) and societal ($26.0 versus $24.7, an incremental cost of $1.3) perspectives (Table 4). In the incremental analysis, prenatal misoprostol distribution had an ICER of US$191 per DALY averted from a government perspective, and US$73 per DALY averted from a modified societal perspective.

**Table 4:** Results of the cost-effectiveness analysis (cost per life saved and cost per DALYs averted). Values in brackets are 95% Credibility Intervals for incremental costs and outcomes from the PSA.

	Government		Societal	
	No Misoprostol	Misoprostol	No Misoprostol	Misoprostol
Mean costs (US $)	12.3	15.6	24.7	26.0
Incremental costs (US $)	-	3.3 (2.1, 4.2)	-	1.3 (-1.6, 2.8)
Incidence of PPH	5.5%	4.5%	5.5%	4.5%
Change in incidence of PPH		1.0% (0.55%, 1.95%)		1.0% (0.55%, 1.95%)
Mortality	0.36%	0.28%	0.36%	0.28%
Change in mortality	-	0.08% (0.04%, 0.13%)	-	0.08% (0.04%, 0.13%)
ICER (US $/life saved)		4244 (1807, 10104)		1623 (-1886, 5727)
Mean DALYs	0.06	0.08	0.06	0.08
Discounted DALYs averted	-	0.02 (0.01, 0.03)	-	0.02 (0.01, 0.03)
ICER (US $/DALY averted)		191 (83, 443)		73 (-86, 256)

### Scenario analyses

In the stratified analysis (Table 5), there was negative correlation between wealth quintile and incremental costs and outcomes, with a tendency for greatest benefit in the lower wealth quintiles.

**Table 5:** Incremental costs, incremental outcomes and incremental cost-effectiveness ratios stratified by wealth quintile.

Wealth quintile	Incremental costs (US $)		Δ in PPH incidence (%)	Δ in mortality (%)	DALYs averted	ICER (US $/DALY averted)	
	Governmental	Societal				Governmental	Modified Societal
Lowest	0.51	-0.22	0.36	0.027	0.0059	86	Dominant
Second	0.59	0.02	0.30	0.022	0.0048	123	5
Middle	0.64	0.24	0.24	0.017	0.0038	167	64
Fourth	0.60	0.27	0.15	0.011	0.0025	239	109
Highest	0.96	0.94	0.01	0.001	0.0002	4593	4469

In threshold analyses, we found distribution of misoprostol would no longer be cost-effective from government and modified societal perspectives if the probability of complications from misuse exceeded 58% and 59% respectively, the average cost of a misoprostol distribution program exceeded US$42 and US$45 per woman respectively and the cost of collection and destruction of unused doses exceeded US$52 and US$57 per woman respectively. The OR for health facility delivery was an important determinant of the ICERs, displaying a positive correlation i.e., lower (or higher) odds of health facility delivery resulting in lower (or higher) ICERs. The threshold OR at which the ICER crossed zero was 0.48. At this OR, misoprostol distribution becomes cost saving. Conversely, the threshold OR for which the ICER crossed US$1,641 was infinite (S3 Fig).

### Univariate sensitivity analysis

**Fig 2:**
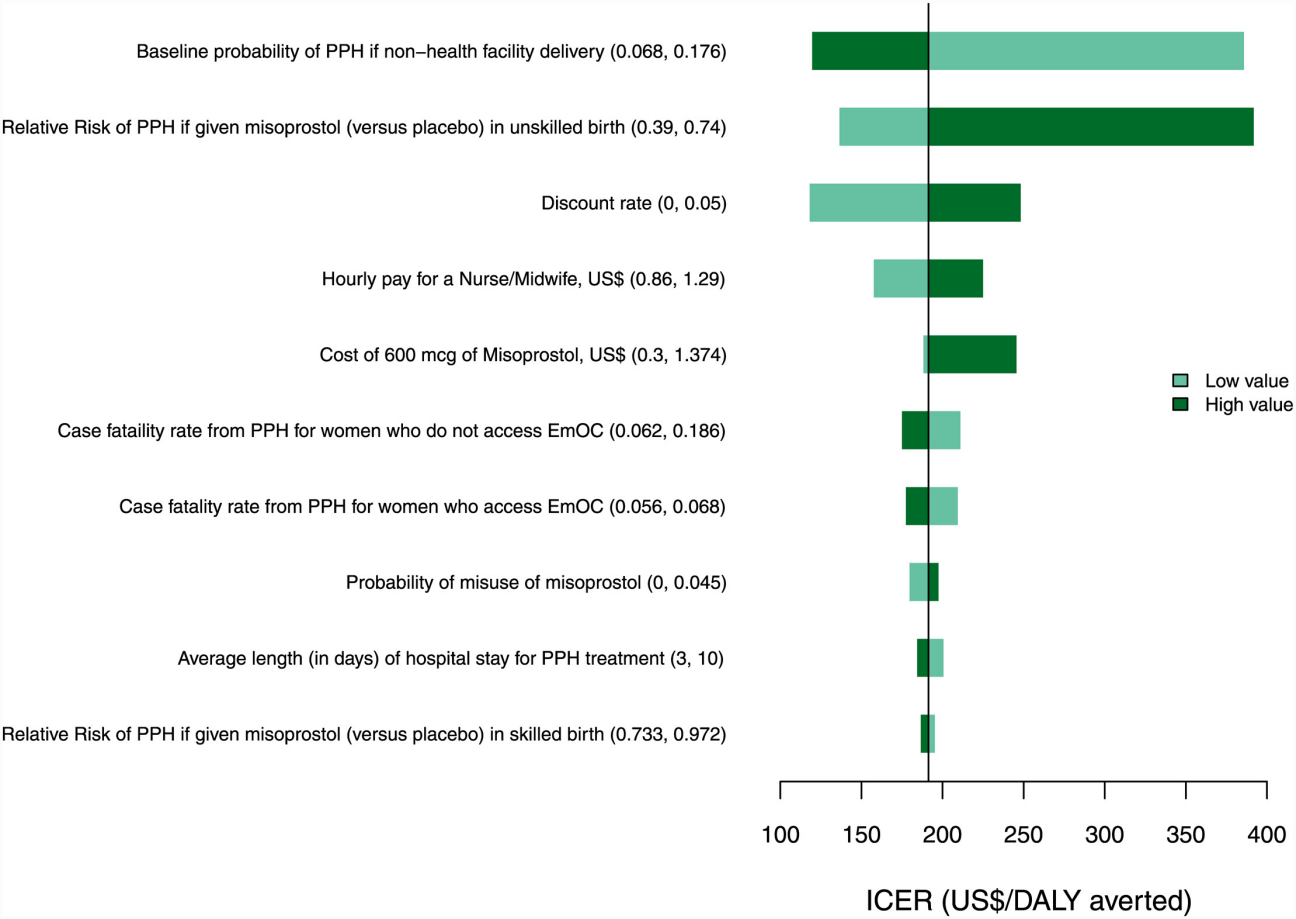
Tornado diagram of univariate sensitivity analysis. The diagram shows the impact of the 10 most influential parameters on the incremental cost per DALY averted from a governmental perspective

**Fig 3:**
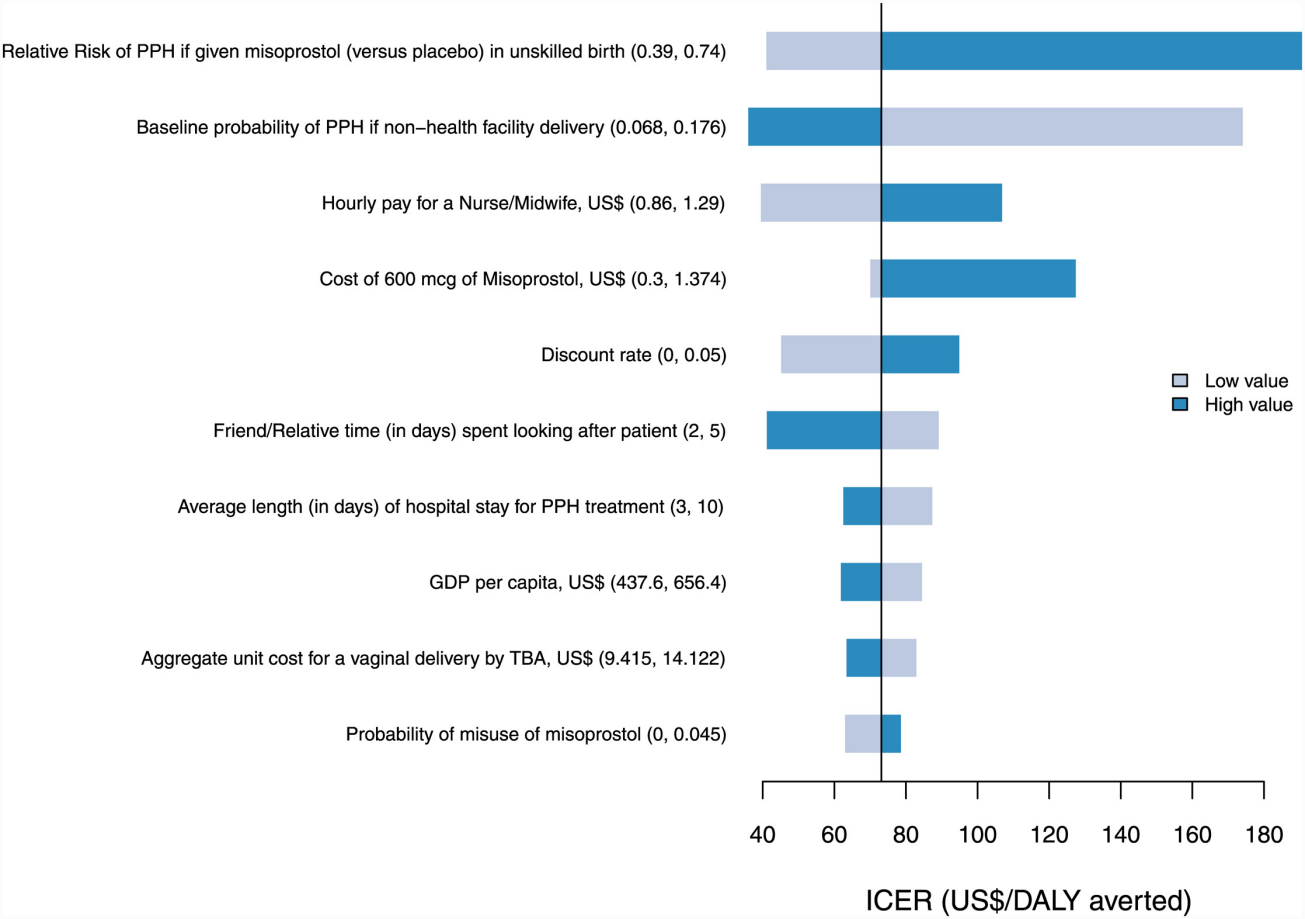
Tornado diagram of univariate sensitivity analysis. The diagram shows the impact of the 10 most influential parameters on the incremental cost per DALY averted from a modified societal perspective

Figs 2 and 3 show the impact of varying individual parameters on the incremental costs and outcomes from both government and modified societal perspectives. ICERs were most sensitive to the baseline incidence of PPH in unskilled deliveries, relative risk of PPH with misoprostol (versus placebo) in unskilled deliveries, the discount rate, the hourly pay for a nurse/midwife and the cost of misoprostol.

### Probabilistic sensitivity analysis

**Fig 4:**
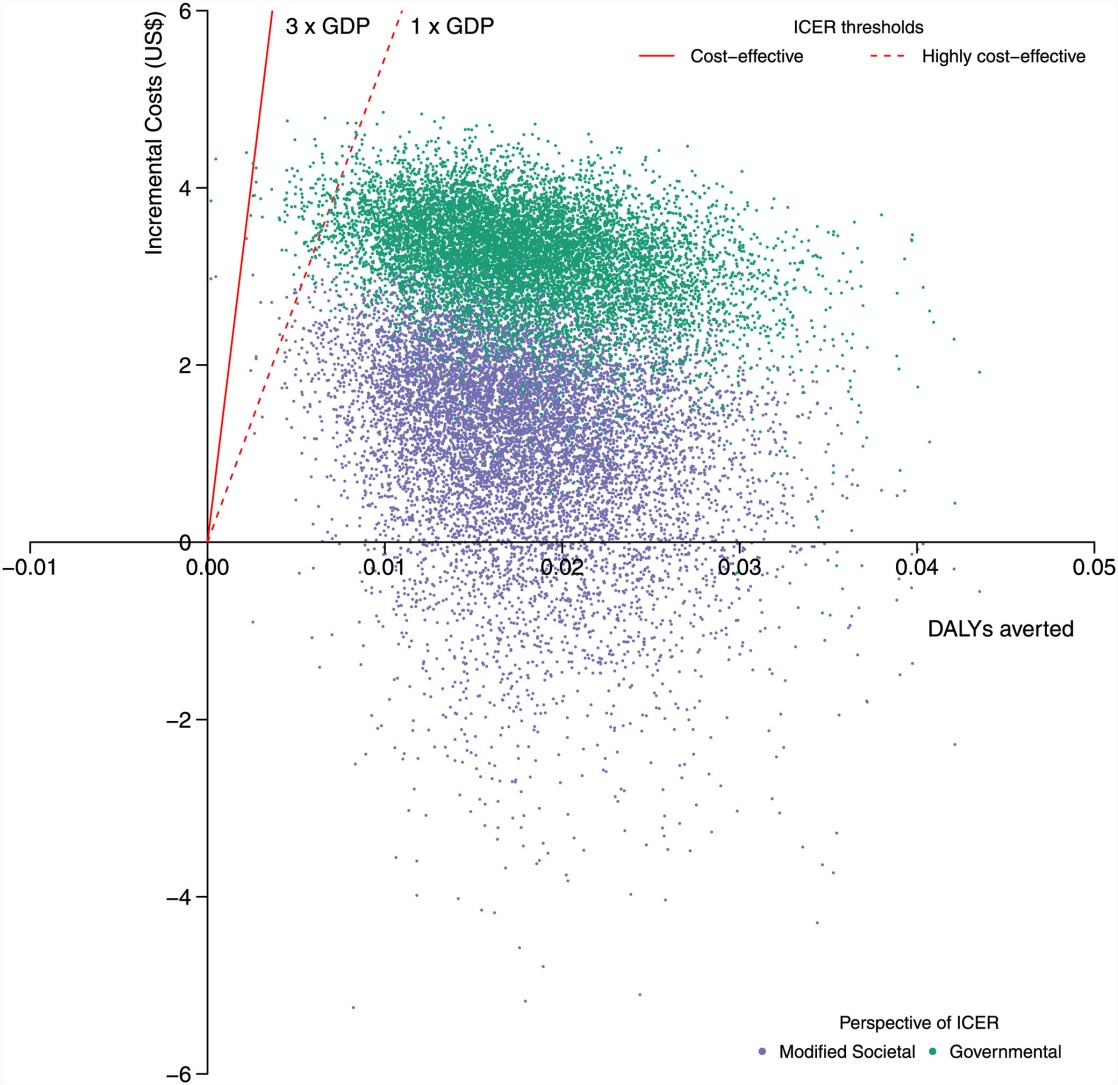
Incremental cost-effectiveness scatter plot showing the distribution of 10,000 incremental cost and DALY averted pairs. The green cloud shows the analysis from the governmental perspective and the purple cloud shows the analysis from the modified societal perspective. The dashed red line represents the lower threshold of willingness to pay per DALY averted (one times the GDP of Uganda) and the solid red line represents the higher threshold of willingness to pay per DALY averted (three times the GDP of Uganda).

**Fig 5:**
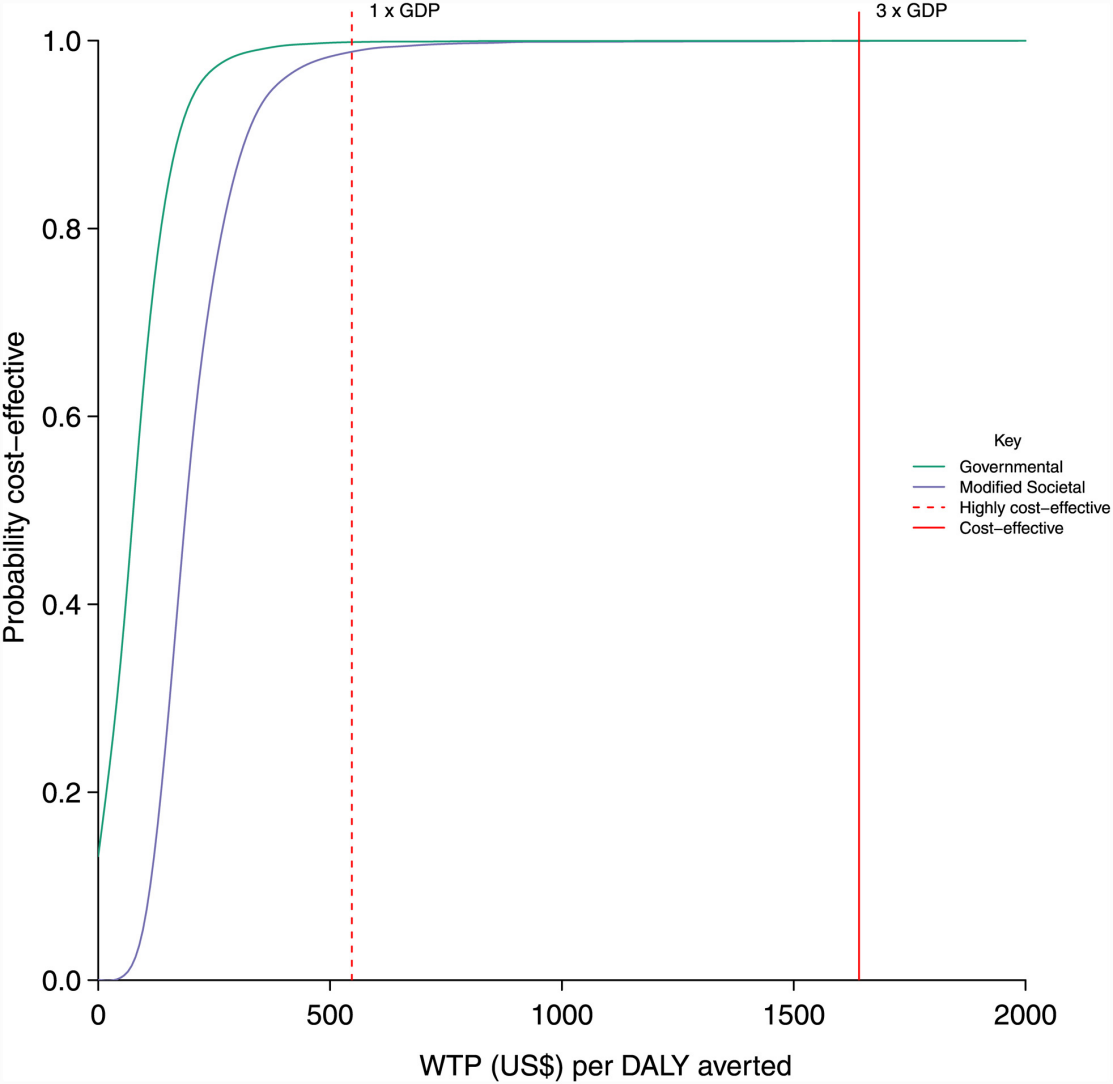
Cost effectiveness acceptability curve obtained from the probabilistic sensitivity analysis. The curves show the proportion of probabilistic iterations (out of 10,000) in which prenatal misoprostol distribution is cost-effective under different thresholds of willingness to pay for a DALY averted. The green curve shows the analysis from the governmental perspective and the purple curve shows the analysis from the modified societal perspective. The dashed red line represents the lower threshold of willingness to pay per DALY averted (one times the GDP of Uganda) and the solid red line represents the higher threshold of willingness to pay per DALY averted (three times the GDP of Uganda)

Results from the PSA are represented as a scatter plot of incremental costs and DALYs averted (Fig 4), as 95% credible intervals in Table 4 and as a CEAC (Fig 5). The reduction in the expected incidence of PPH ranged from 0.06% to 0.15% and the reduction in expected mortality ranged from 0.040% to 0.011%. DALYs averted ranged from 0.01 to 0.03. The increase in mean costs ranged from $2.1 to $4.2 from the government and $-1.6 to $2.8 from the societal perspective. The range on the ICERs were $82 to $443 per DALY averted from the government and $-86 to $256 per DALY averted from the societal perspective. The CEAC (Fig 5) showed that, at a threshold willingness to pay of US$1641 per DALY averted, the prenatal misoprostol distribution was cost-effective in 100% iterations from the governmental and modified societal perspectives.

## Discussion

### Main findings

We used decision analytic methods to examine the potential value of prenatal distribution of misoprostol (as part of safe delivery kits) as a strategy to increase access to uterotonics for the prevention of PPH. We projected that this strategy would decrease the incidence of PPH, mortality and result in fewer DALYs. From the government perspective, these benefits would be achieved at an incremental cost of US$3.3 per mother and this result was robust over the range of parameter values in our sensitivity analyses. From a modified societal perspective, we projected that these benefits would be achieved at an incremental cost of US$1.3 per mother; our sensitivity analyses indicated that prenatal misoprostol distribution ranged from being cost saving and DALY averting to having an ICER of US$256 per DALY averted. We projected that, from both perspectives, the strategy had high probability of being cost-effective at willingness to pay per DALY averted below the WHO recommended threshold for highly cost-effective strategies [51].

### Strengths and limitations

Previous analyses have focused on home births [12,13] or births attended by TBAs [14]. Our analysis considers a more likely scenario in developing countries in which the delivery path a woman could take is uncertain. We incorporate oxytocin as the first line of treatment for patients who deliver in settings where it is available and only advocate misoprostol as a second line drug. This is in line with current WHO/FIGO guidelines for prevention of PPH [4,19]. Unlike previous analyses [12–14], we account for a possibility of externalities of misoprostol use (changes in maternal health seeking behavior) and adverse consequences of misuse (uterine rupture and stillbirth), and examine in separate analyses the threshold values of these externalities that would negate the potential value of this strategy. We borrow from the analysis of Pagel et al. [59] to account for the differences in health facility delivery rates by socioeconomic status, and extend their analysis by examining the potential impact on both costs and outcomes. We used the most recent estimates from meta-analyses of randomized studies of the different uterotonics, which should lend credence to our analyses. Costs were measured from both governmental and modified societal perspectives thus providing a more holistic picture of the strategy’s potential value.

There are several limitations of our analysis. While we advocate for SDKs as a potential avenue for distribution of misoprostol, stock-outs of the freely available SDKs at public health facilities may adversely affect access to misoprostol distributed this way. A dual strategy where misoprostol is distributed to mothers at prenatal visits (without SDKs) could be considered. We made a key assumption that misoprostol would not be misused as an abortifacient, either by the mother herself, or by a woman to whom she could sell the drug. While this assumption potentially favors prenatal misoprostol distribution in our analysis, we argue that demonstration projects in Africa show that this type of misuse is unlikely [15,16]. This is plausible given extensive health education and limiting distribution to mothers who are further along in their pregnancies; for instance the *MamaMiso* study in Uganda recruited women more than 34 weeks of gestation and provided extensive health education [18]. We envisage that this is how such an intervention would be deployed.

We made optimistic assumptions about the coverage rate of misoprostol both in health facility and non-health facility deliveries. However, these are borrowed from a project in Tanzania [33] that is similar to Uganda both culturally and in maternal health seeking behavior. Our base case assumes that such a program would not positively incentivize health facility delivery, or adversely incentivize home deliveries (at the expense of health facility delivery). The reality is that either is a possibility, although evidence suggests a positive effect [15,17]. We therefore examined, in threshold and scenario analyses, the impact of a change in odds of health facility delivery induced by misoprostol distribution. We projected that increases in odds of health facility delivery make misoprostol distribution “*less cost-effective*” (higher ICERs), while decreasing odds of health facility delivery make it “*more cost-effective*” (lower ICERs). Incentivizing health facility delivery itself could improve outcomes, potentially making misoprostol distribution less efficient. Policy makers need to understand this dynamic effect as they debate the merits of this strategy. However, we projected that, even with very large increases in odds of health facility delivery, misoprostol would still be cost effective. Policy makers and implementers could maximize efficiency by both encouraging facility delivery, and distribution of misoprostol.

Finally, we do not account for important potential outcomes that may have biased our results. We assumed that all women would undergo a normal delivery. This was necessitated by a desire to simplify the model (to enhance usefulness) and a lack of local data. This could have biased the results in favor of misoprostol, because home deliveries that turn out to be complicated (needing cesarean delivery), could have potentially worse outcomes and higher costs. Unfortunately the modelling framework is not set up to examine this in scenario or sensitivity analyses therefore this is an area of future work to improve our modelling framework. Prenatal misoprostol distribution may reduce the incentive for hospital managers to maintain order and re-supply quantities of oxytocin at health facilities or could lead to clinicians reserving oxytocin for treatment of PPH, in which case the probability that a woman receives misoprostol for prevention of PPH would reduce. However, changes in this parameter did not substantially influence our results.

### Interpretation

Our model predicted that prenatal distribution of misoprostol is a potentially valuable strategy for prevention of PPH in this setting. Three previous studies have estimated the potential cost-effectiveness of misoprostol for the prevention of PPH. Sutherland et al. [13] and Sutherland and Bishai [12], showed misoprostol was cost-effective for prevention of PPH among women who give birth at home in India. Bradley et al. [14] showed that misoprostol was a dominant strategy when compared to simple referral for EmOC for births attended by TBAs in Tanzania. We extend these results to project that prenatal distribution of misoprostol (irrespective of predicted place of delivery) is a potentially cost effective strategy. Further, it would target economically productive women (mothers). Therefore, there are potential added benefits in terms of increased household productivity and averted household medical expenditure. A general message from these, and our study is that prenatal distribution of misoprostol could save lives of mothers during birth, contributing towards Millennium Development Goal 5 (MDG 5) [60].

Prenatal misoprostol distribution appears to be a good buy when compared to other interventions which are currently implemented in Uganda, such as short course amphotericin (7 days) plus high dose fluconazole (1200mg/day) versus high dose fluconazole monotherapy ($15.11/QALY gained) for *Cryptococcal* meningitis in HIV patients [61], and early versus delayed initiation of HAART ($460/DALY averted) [62]. It compares favorably with other interventions targeting maternal health e.g., universal access to contraceptives, which both save lives and save costs [58].

There are a number of important parameters to which our results are sensitive. The relative risk of PPH with misoprostol (versus placebo) in unskilled births and skilled births has long been a subject of significant debate in the medical literature [17,63]. On appraising the different analyses, we take the view that, at least where AMTSL is not an option because of absence of skilled attendance at delivery, misoprostol is beneficial in preventing PPH. Ethical issues may preclude more randomized controlled trials to definitively answer the question of the effectiveness of misoprostol in different settings although observational studies could examine the occurrence of PPH in women who receive preventive misoprostol particularly in lower level health facilities where there is a paucity of skilled attendants. The Global Burden of Diseases study, 2010 excluded time discounting of health benefits [2]. In our analysis, DALYs averted were higher and consequently prenatal distribution was more cost effective (ICERs were lower) when we did not discount future YLL. We model a wide range of costs of a 600mcg dose of misoprostol because of uncertainty over the actual public sector procurement price.

The country-specific incidence of PPH is poorly described. A higher incidence of PPH could be potentially associated with cost savings and more DALYs averted. Indeed, many incident PPH cases go unnoticed because many mothers give birth at home and are unable to access health care. In this scenario, our analysis underestimates the value of misoprostol distribution. Studies are needed to improve local estimates of incidence and outcomes of PPH. Case fatality rates from PPH are described in the literature [23,42], but the studies do not differentiate deaths by place of delivery, this is key to understanding the value of prenatal misoprostol distribution. If case fatality rates were higher, our analysis would underestimate the value of prenatal misoprostol distribution. Lastly, we had no robust data on the probability of misuse and the conditional probabilities of the consequences of misuse of misoprostol, yet these are potentially important parameters in estimating the value of misoprostol distribution. If countries move towards adopting this strategy, there is a need to conscientiously monitor misuse and its consequences.

There are important questions around maternal health interventions for PPH prevention that remain unanswered: 1) whether ensuring oxytocin availability at all health facilities represents better value for money, 2) whether targeting women who are less likely to attend health facilities during labor would be more efficient, 3) whether strategies that increase the proportion of women delivering in health facilities would be better value for money, and 4) the incremental value of using a combination of these approaches. While these are interesting questions, the interventions they suggest may not be feasibly implemented in the short run in a resource poor environment and are therefore better looked at as longer-term strategies. For instance ensuring oxytocin availability would require a functional nationwide cold chain supply system, strategies for improving health facility birth rates are still poorly understood, and good models for predicting which women are unlikely to deliver in health facilities are non-existent. There are feasible technologies e.g., Oxytocin in Uniject devices that could be added to this model as a comparator [34]. These represent potential areas of future research towards achieving MDG 5 [60]. By disaggregating the incremental costs and benefits by wealth quintile in the scenario analysis, we projected that prenatal misoprostol distribution is likely to benefit women in the lower wealth quintiles more, primarily because they are less likely to attend health facilities for delivery. This suggests that if we were better able to predict which mothers were unlikely to deliver in health facilities, this intervention could then be targeted towards them.

## Conclusion

Prenatal distribution of misoprostol could potentially save lives and at modest incremental costs in this setting. Policy makers should consider this as a strategy towards meeting the targets of reductions in maternal morbidity and mortality in MDG 5 [60].

## Supporting Information

S1 Table Health resource use estimates and unit costs for the intervention (community distribution of misoprostol) and outcomes—vaginal delivery, postpartum hemorrhage, uterine rupture and still birth—in the model (DOCX)

S2 Table Summary of cost estimates for the outcomes—vaginal delivery, postpartum hemorrhage, uterine rupture and still birth—in the model. Vaginal delivery costs are calculated according to delivery pathway trajectory
(DOCX)

S3 Table Misoprostol program costing model (DOCX)

S1 Fig A random effects meta-analysis of the incidence of PPH derived from the non-interventional arm of four studies comparing uterotonics to no uterotonic in which expectant management of the third stage of labor was practiced (EPS)

S2 Fig A random effects meta-analysis of the relative risk of PPH from two trials comparing misoprostol to placebo where active management of the third stage of labor was practiced (EPS)

S3 Fig Impact of changes in odds ratio of health facility delivery on the ICERs from governmental (green curve) and modified societal (purple curve) perspectives (EPS)
