# Correction: Potential Cost-Effectiveness of Prenatal Distribution of Misoprostol for Prevention of Postpartum Hemorrhage in Uganda

**DOI:** 10.1371/journal.pone.0152955

**Published:** 2016-03-31

**Authors:** Solomon J. Lubinga, Esther C. Atukunda, George Wasswa-Ssalongo, Joseph B. Babigumira

There are errors in the sixth and eighth sentences of the “Methods and Findings” section of the Abstract. The correct sentences are “In the base-case analysis, misoprostol distribution lowered the expected incidence of PPH by 1.2% (95% credibility interval (CrI): 0.55%, 1.95%), mortality by 0.08% (95% CrI: 0.04%, 0.13%) and DALYs by 0.02 (95% CrI: 0.01, 0.03).” and “ICERs were US$181 (95% CrI: 81, 443) per DALY averted from a governmental perspective, and US$64 (95% CrI: -84, 260) per DALY averted from a modified societal perspective.”

There are errors in the third sentence of the “Misuse and potential changes in delivery pathway trajectory following misoprostol distribution” section of the Methods. The correct sentence is “In Uganda, the MamaMiso study found that 99.7% of women used misoprostol appropriately, and the 0.3% that took it after delivery show no adverse events [18].”

There are errors in the first and fifth sentences of the “Cost-effectiveness analysis” subsection of the Results. The correct sentences are “In the base-case, the expected incidence of PPH was lower with prenatal misoprostol distribution (4.3% versus 5.5%; an absolute reduction of 1.2% and relative reduction of 28.7%).” and “In the incremental analysis, prenatal misoprostol distribution had an ICER of US$181 per DALY averted from a government perspective, and US$64 per DALY averted from a modified societal perspective.”

There are errors in the fifth sentence of the “Probabilistic sensitivity analysis” section of the Results. The correct sentence is “The range on the ICERs were $81 to $441 per DALY averted from the government and $-84 to $260 per DALY averted from the societal perspective.”

There are errors in Tables 4 and 5. Please see the corrected Tables 4 and 5 here.

**Table 4:** Results of the cost-effectiveness analysis (cost per life saved and cost per DALYs averted). Values in brackets are 95% Credibility Intervals for incremental costs and outcomes from the PSA.

	Government		Societal	
	No Misoprostol	Misoprostol	No Misoprostol	Misoprostol
Mean costs (US $)	12.3	15.6	24.7	26.0
Incremental costs (US $)	-	3.3 (2.1, 4.2)	-	1.3 (-1.6, 2.8)
Incidence of PPH	5.5%	4.3%	5.5%	4.3%
Change in incidence of PPH		1.2% (0.55%, 1.96%)		1.2% (0.55%, 1.96%)
Mortality	0.36%	0.28%	0.36%	0.28%
Change in mortality	-	0.08% (0.04%, 0.13%)	-	0.08% (0.04%, 0.13%)
ICER (US $/life saved)		4010 (1798, 9998)		1418 (-1952, 5662)
Mean DALYs	0.06	0.08	0.06	0.08
Discounted DALYs averted	-	0.02 (0.01, 0.03)	-	0.02 (0.01, 0.03)
ICER (US $/DALY averted)		181 (81, 441)		64 (-84, 260)

**Table 5:** Incremental costs, incremental outcomes and incremental cost-effectiveness ratios stratified by wealth quintile.

Wealth	Incremental costs (US $)		Δ in PPH	Δ in mortality	DALYs	ICER (US $/DALY averted)	
quintile	Governmental	Societal	incidence (%)	(%)	averted	Governmental	Modified Societal
Lowest	0.49	-0.26	0.40	0.028	0.0061	80	Dominant
Second	0.57	-0.01	0.33	0.022	0.0050	115	Dominant
Middle	0.63	0.22	0.26	0.018	0.0040	158	56
Fourth	0.59	0.26	0.17	0.012	0.0026	226	97
Highest	0.96	0.93	0.02	0.001	0.0002	3947	3836

There are errors in Figs 2, 3, 4 and 5 and S3 Fig. Please see the corrected Figs 2, 3, 4 and 5 and S3 Fig here.

**Fig 2:**
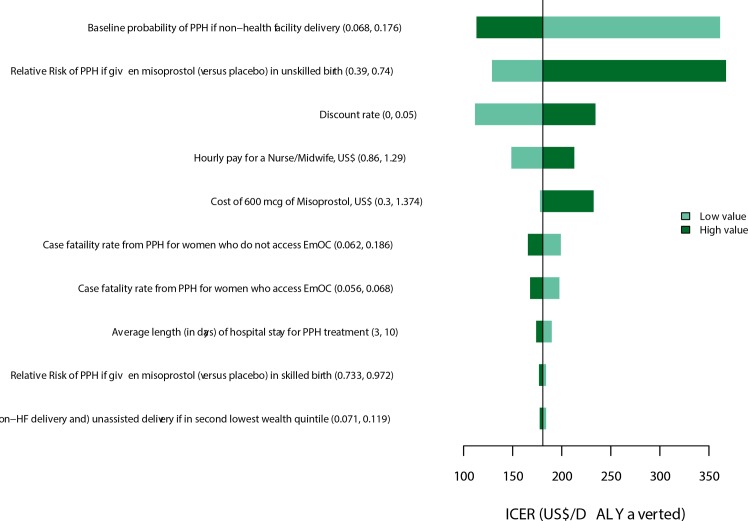
Tornado diagram of univariate sensitivity analysis. The diagram shows the impact of the 10 most influential parameters on the incremental cost per DALY averted from a governmental perspective.

**Fig 3:**
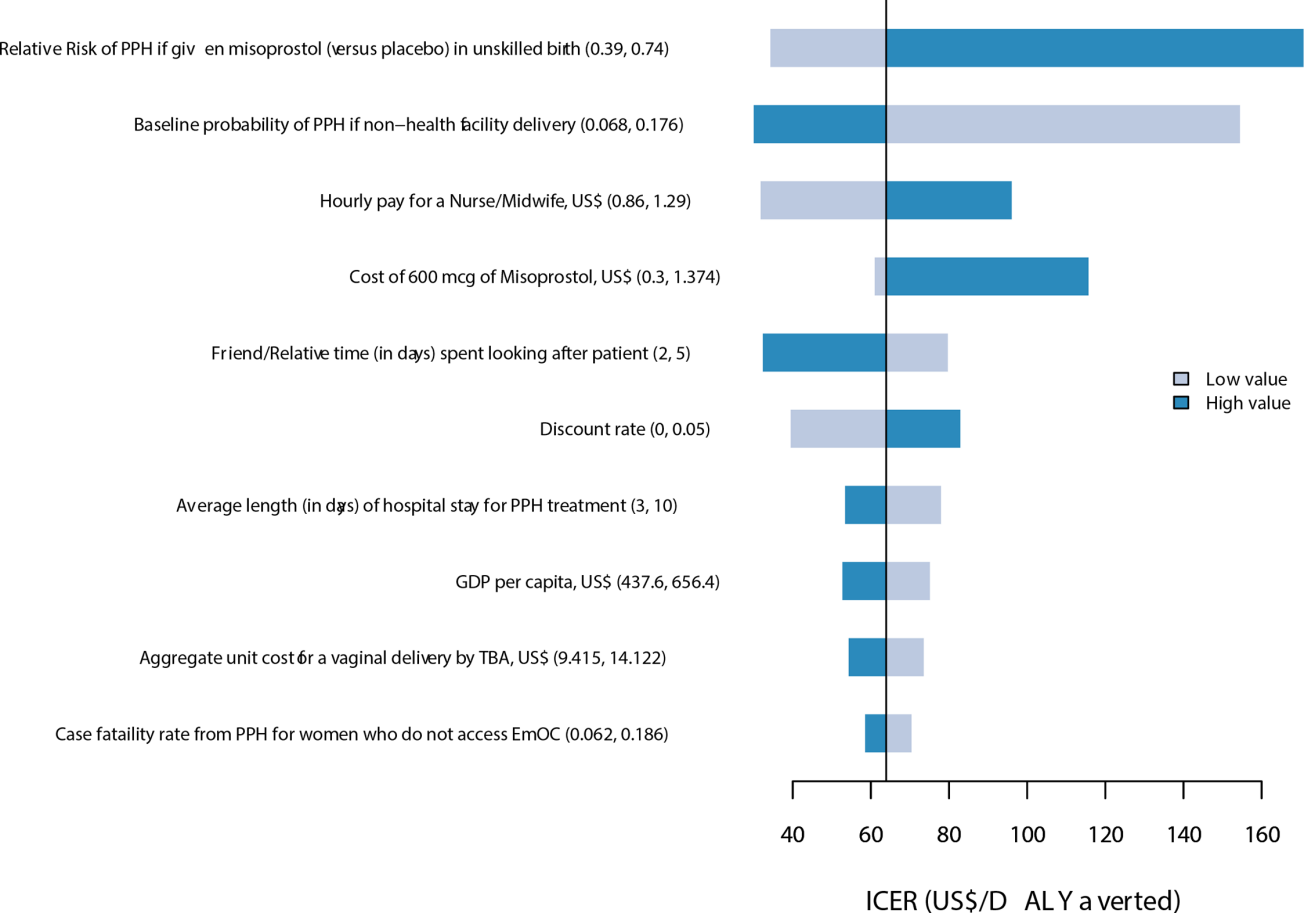
Tornado diagram of univariate sensitivity analysis. The diagram shows the impact of the 10 most influential parameters on the incremental cost per DALY averted from a modified societal perspective.

**Fig 4:**
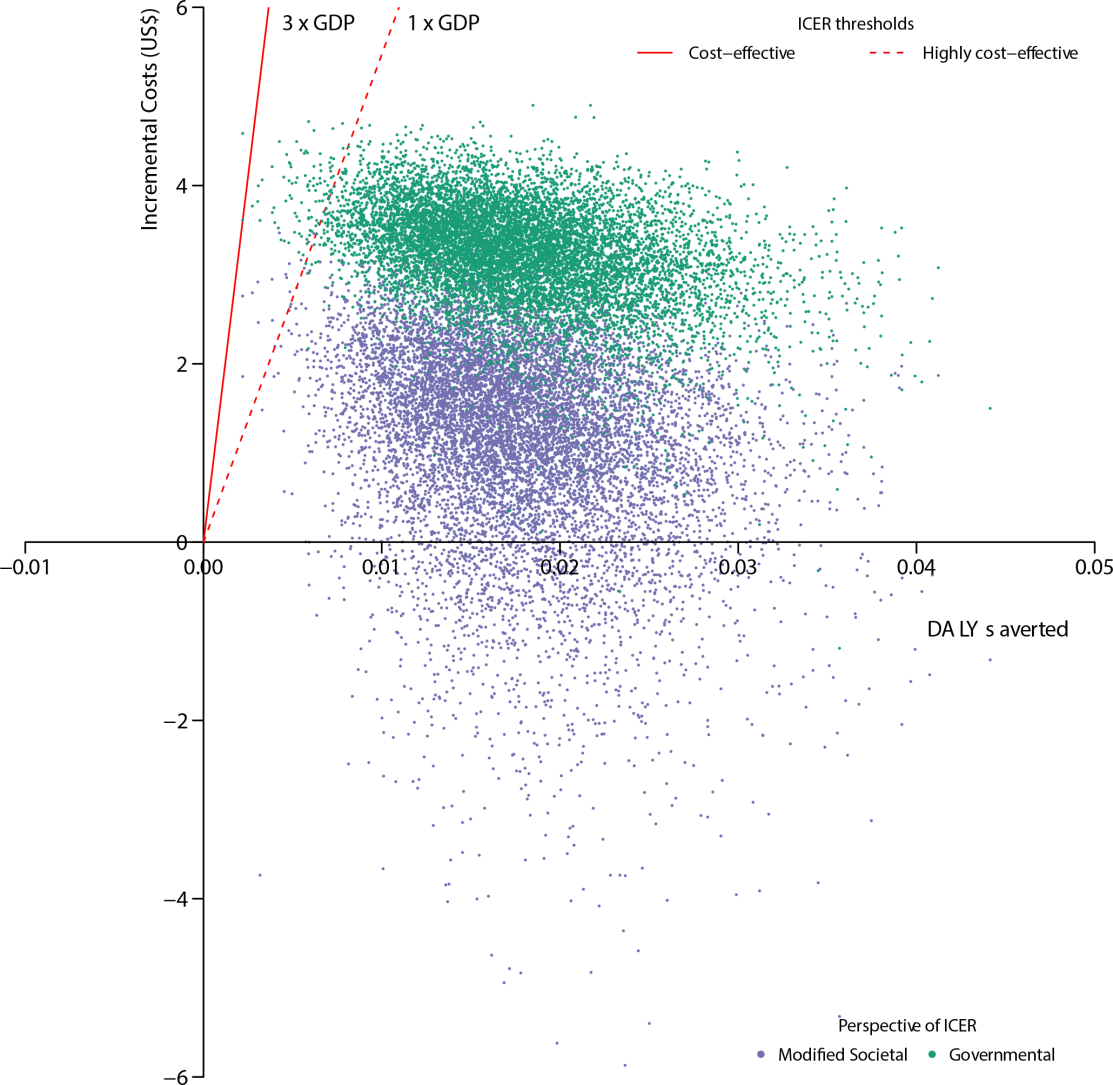
Incremental cost-effectiveness scatter plot showing the distribution of 10,000 incremental cost and DALY averted pairs. The green cloud shows the analysis from the governmental perspective and the purple cloud shows the analysis from the modified societal perspective. The dashed red line represents the lower threshold of willingness to pay per DALY averted (one times the GDP of Uganda) and the solid red line represents the higher threshold of willingness to pay per DALY averted (three times the GDP of Uganda).

**Fig 5:**
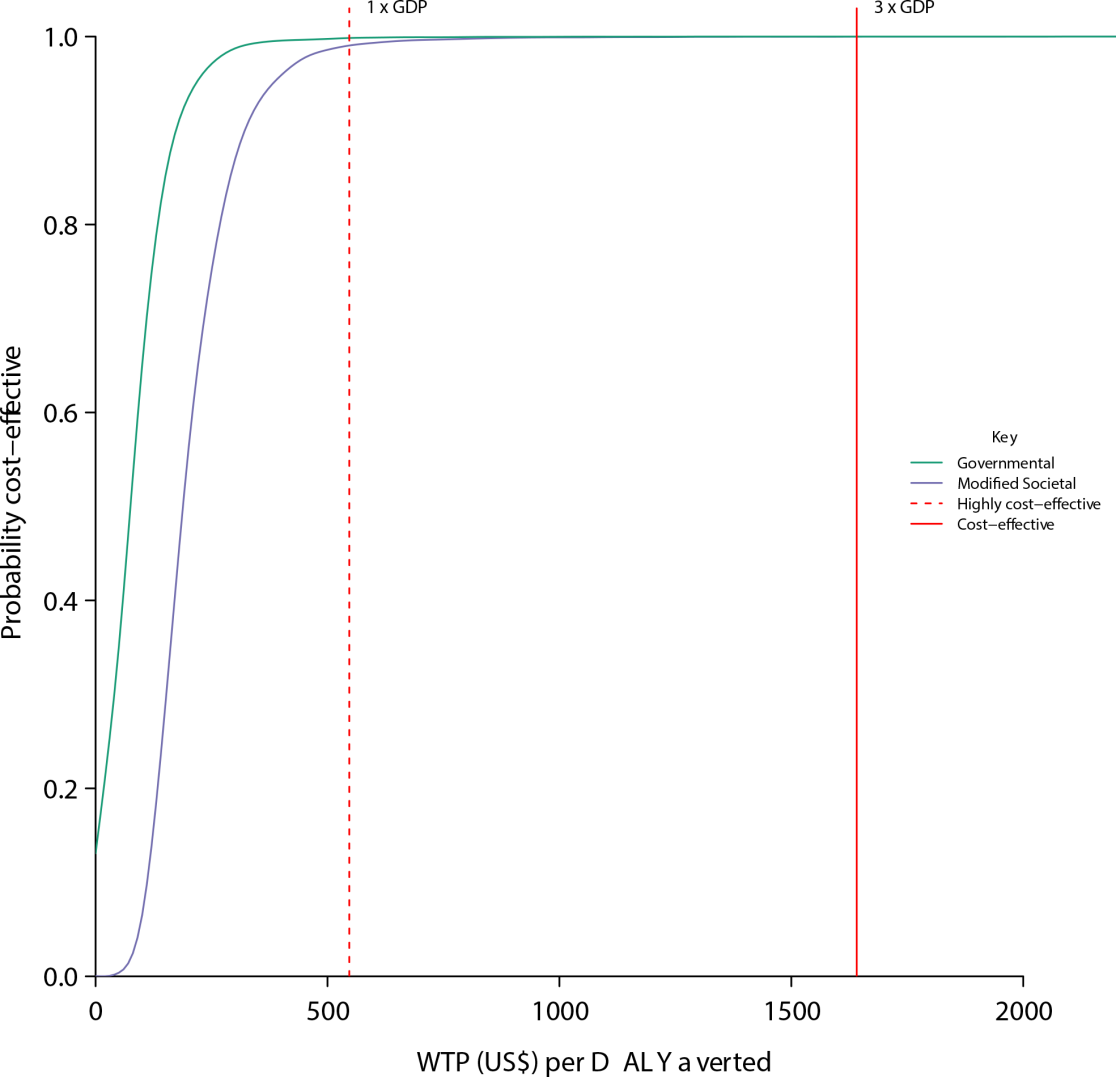
Cost effectiveness acceptability curve obtained from the probabilistic sensitivity analysis. The curves show the proportion of probabilistic iterations (out of 10,000) in which prenatal misoprostol distribution is cost-effective under different thresholds of willingness to pay for a DALY averted. The green curve shows the analysis from the governmental perspective and the purple curve shows the analysis from the modified societal perspective. The dashed red line represents the lower threshold of willingness to pay per DALY averted (one times the GDP of Uganda) and the solid red line represents the higher threshold of willingness to pay per DALY averted (three times the GDP of Uganda).

## Supporting Information

S3 Fig Impact of changes in odds ratio of health facility delivery on the ICERs from governmental (green curve) and modified societal (purple curve) perspectives. (EPS)
