# Supplementary material for: Potential Cost-Effectiveness of Prenatal Distribution of Misoprostol for Prevention of Postpartum Hemorrhage in Uganda
Source: PLoS One. 2015 Nov 11;10(11):e0142550. doi: 10.1371/journal.pone.0142550 (PMC4641649; doi:10.1371/journal.pone.0142550)
Supplement: S1 Table — (DOCX) [file pone.0142550.s001.docx]

S1 Table. Health resource use estimates and unit costs for the intervention (community distribution of misoprostol) and outcomes—vaginal delivery, postpartum hemorrhage, uterine rupture and stillbirth—in the model

| **Variable** | **Base case** | **Low value** | **High value** | **Distribution** | **Reference** |
| --- | --- | --- | --- | --- | --- |
| *Unit Costs ($)* |  |  |  |  |  |
| Lidocaine 2% inj, 50mL | 0.47 | 0.37 | 0.56 | Normal | NMS [1] |
| Povidone Iodine solution | 1.03 | 0.82 | 1.23 | Normal | NMS [1] |
| Ferrous salt + Folic acid tablets 60mg + 0.25mg | 0.00 | 0.00 | 0.00 | Normal | NMS [1] |
| Paracetamol tablets 500mg | 0.00 | 0.00 | 0.00 | Normal | NMS [1] |
| Tetracycline eye ointment, 1% | 0.18 | 0.15 | 0.22 | Normal | NMS [1] |
| Vitamin A capsules, 200,000IU | 0.03 | 0.03 | 0.04 | Normal | NMS [1] |
| Maternity bed-day at a Health Center | 3.67 | 2.94 | 4.41 | Normal | Adam *et al.* [2] |
| Maternity bed-day at a Hospital | 3.83 | 3.07 | 4.60 | Normal | Adam *et al.* [2] |
| Mucus Extractor, infant | 0.51 | 0.41 | 0.61 | Normal | NMS [1] |
| Gauze, Sterile | 0.01 | 0.00 | 0.01 | Normal | NMS [1] |
| Gloves, Sterile | 0.23 | 0.19 | 0.28 | Normal | NMS [1] |
| Suture catgut, obstetric, sterile, with needle | 1.15 | 0.92 | 1.38 | Normal | NMS [1] |
| AD Syringe, 5mL disposable + needle | 0.06 | 0.04 | 0.07 | Normal | NMS [1] |
| AD Syringe, 10mL disposable + needle | 0.10 | 0.08 | 0.12 | Normal | NMS [1] |
| Umbilical tape | 0.10 | 0.08 | 0.12 | Normal | NMS [1] |
| Delivery record | 0.52 | 0.42 | 0.62 | Normal | NMS [1] |
| Blood, one unit | 27.86 | 22.29 | 33.43 | Normal | Lara *et al.* [3] |
| Ampicillin, injection 1g | 0.05 | 0.04 | 0.05 | Normal | NMS [1] |
| Diazepam 5mg/ml, injection 2mL | 0.09 | 0.07 | 0.11 | Normal | NMS [1] |
| Doxycycline, tablet 100mg | 0.02 | 0.01 | 0.02 | Normal | NMS [1] |
| Gentamicin 40 mg/ml, injection 2mL | 0.05 | 0.04 | 0.06 | Normal | NMS [1] |
| Halothane gas, Inhalation | 23.99 | 19.19 | 28.79 | Normal | NMS [1] |
| Metronidazole 5mg/mL, injection 100mL | 0.34 | 0.27 | 0.41 | Normal | NMS [1] |
| Pethidine 50mg/mL, injection 1mL | 0.63 | 0.50 | 0.75 | Normal | NMS [1] |
| Sodium lactate (Ringer) + set, solution 500mL | 0.49 | 0.39 | 0.58 | Normal | NMS [1] |
| Suxamthonium 50mg/mL, injection 2mL | 0.57 | 0.46 | 0.69 | Normal | NMS [1] |
| Thiopental, injection 1g | 1.67 | 1.33 | 2.00 | Normal | NMS [1] |
| Blood group test | 1.07 | 0.85 | 1.28 | Normal | Ninci and Ocakacon [4] |
| Haemoglobin test | 0.75 | 0.60 | 0.89 | Normal | Ninci and Ocakacon [4] |
| Hourly pay for a Medical Officer | 2.94 | 2.35 | 3.53 | Normal | Matsiko and Kiwanuka [5] |
| Hourly pay for an Anesthetist | 1.36 | 1.09 | 1.63 | Normal | Matsiko and Kiwanuka [5] |
| Hourly pay for an Obstetrician | 5.02 | 4.01 | 6.02 | Normal | Matsiko and Kiwanuka [5] |
| Hourly pay for a Nurse/Midwife | 1.07 | 0.86 | 1.29 | Normal | Matsiko and Kiwanuka [5] |
| Hourly pay for a Nursing aide | 0.83 | 0.66 | 0.99 | Normal | Matsiko and Kiwanuka [5] |
| Blood giving set with needle, disposable | 0.47 | 0.37 | 0.56 | Normal | NMS [1] |
| Blood lancet, disposable | 0.06 | 0.05 | 0.07 | Normal | NMS [1] |
| Catheter, foley | 0.44 | 0.35 | 0.53 | Normal | NMS [1] |
| Infusion giving set with needle, disposable | 0.13 | 0.10 | 0.16 | Normal | NMS [1] |
| Nitrous oxide, 100 liters | 478.25 | 382.60 | 573.90 | Normal | NMS [1] |
| Oxygen, percubic meter | 2.64 | 1.70 | 5.87 | Normal | MSH [6] |
| Suture needle, assorted sizes, round body | 6.33 | 5.07 | 7.60 | Normal | NMS [1] |
| Water for injection, 5mL | 0.03 | 0.02 | 0.04 | Normal | NMS [1] |
| Oxytocin 10IU/1mL injection | 0.04 | 0.04 | 0.05 | Normal | NMS [1] |
| 600 mcg dose of Misoprostol (200mcg caps) | 0.85 | 0.63 | 1.45 | Normal | MSH [6] |
| Aggregate cost for a vaginal delivery by private midwife | 12.28 | 9.82 | 14.73 | Normal | Levin *et al.* [7] |
| Aggregate cost for a vaginal delivery by TBA | 11.77 | 9.41 | 14.12 | Normal | Levin *et al.* [7] |
| Friend/Relative time (in days) spent looking after patient | 3.00 | 1.00 | 5.00 | Exponential | Assumed |
| Average travel costs to Hospital | 10.22 | 8.17 | 12.26 | Normal | Levin *et al.* [7] |
| Average travel to Health Center | 1.39 | 1.11 | 1.66 | Normal | Levin *et al.* [7] |
| Upkeep costs at Hospital | 8.08 | 6.47 | 9.70 | Normal | Levin *et al.* [7] |
| Upkeep costs at Health Center | 2.77 | 2.22 | 3.33 | Normal | Levin *et al.* [7] |
| GDP per capita | 547.00 | 437.60 | 656.40 | Normal | World Bank, 2012 |
| Collection and destruction of unused misoprostol | 0.05 | 0.04 | 0.06 | Normal | Assumed |
|  |  |  |  |  |  |
| ***Health resource use for PPH management*** |  |  |  |  |  |
| Anesthetist hours per patient | 1.00 | 0.50 | 1.50 | Exponential | Weissman *et al.* [8] |
| Auxiliary health worker hours per patient | 4.50 | 2.25 | 6.75 | Exponential | Weissman *et al.* [8] |
| Nurse/midwife hours per patient | 2.00 | 1.00 | 3.00 | Exponential | Weissman *et al.* [8] |
| Obstetrician hours per patient | 1.00 | 0.50 | 1.50 | Exponential | Weissman *et al.* [8] |
| Average length (in days) of hospital stay | 7.00 | 3.50 | 10.50 | Exponential | Weissman *et al.* [8] |
| Average travel time (in hours) to a hospital | 0.75 | 0.38 | 1.13 | Normal | Weissman *et al.* [8] |
|  |  |  |  |  |  |
| *Proportion of PPH patients utilizing resource* |  |  |  |  |  |
| Anesthesia | 0.20 | 0.10 | 0.30 | Beta | Weissman *et al.* [8] |
| Blood transfusions | 0.10 | 0.05 | 0.15 | Beta | Weissman *et al.* [8] |
| Ampicillin, injection 1g | 0.50 | 0.25 | 0.75 | Beta | Weissman *et al.* [8] |
| Diazepam 5mg/ml, injection 2ml | 0.80 | 1.00 | 1.00 | Beta | Weissman *et al.* [8] |
| Doxycycline, tablet 100mg | 0.50 | 0.25 | 0.75 | Beta | Weissman *et al.* [8] |
| Gentamicin 40 mg/ml, injection 2ml | 0.50 | 0.25 | 0.75 | Beta | Weissman *et al.* [8] |
| Halothane gas | 0.20 | 0.10 | 0.30 | Beta | Weissman *et al.* [8] |
| Lidocaine 1%, injection 50ml | 0.50 | 0.25 | 0.75 | Beta | Weissman *et al.* [8] |
| Metronidazole 5mg/ml, injection 100ml | 0.50 | 0.25 | 0.75 | Beta | Weissman *et al.* [8] |
| Oxytocin 10 IU/ml, injection 1ml | 1.00 | 1.00 | 1.00 | Beta | Weissman *et al.* [8] |
| Paracetamol, tablets 500mg | 0.50 | 0.25 | 0.75 | Beta | Weissman *et al.* [8] |
| Pethidine 50mg/ml, injection 1ml | 0.75 | 0.38 | 1.13 | Beta | Weissman *et al.* [8] |
| Sodium lactate (Ringer) + set, solution 500ml | 0.20 | 0.10 | 0.30 | Beta | Weissman *et al.* [8] |
| Sodium lactate (Ringer) + set, solution 500ml | 1.00 | 1.00 | 1.00 | Beta | Weissman *et al.* [8] |
| Suxamthonium 50mg/ml, injection 2ml | 0.20 | 0.10 | 0.30 | Beta | Weissman *et al.* [8] |
| Thiopental, injection 1g | 0.20 | 0.10 | 0.30 | Beta | Weissman *et al.* [8] |
| Blood giving set with needle, disposable | 0.10 | 0.05 | 0.15 | Beta | Weissman *et al.* [8] |
| Blood lancet, disposable | 1.00 | 1.00 | 1.00 | Beta | Weissman *et al.* [8] |
| Catheter, foley | 1.00 | 1.00 | 1.00 | Beta | Weissman *et al.* [8] |
| Gauze pad, sterile, 12ply 76x76 | 0.50 | 0.25 | 0.75 | Beta | Weissman *et al.* [8] |
| Gloves, surgeons, disposable, pair | 1.00 | 1.00 | 1.00 | Beta | Weissman *et al.* [8] |
| Infusion giving set with needle, disposable | 1.00 | 1.00 | 1.00 | Beta | Weissman *et al.* [8] |
| Oxygen for general anesthesia | 0.20 | 0.10 | 0.30 | Beta | Weissman *et al.* [8] |
| In shock who require Oxygen (5%, 15%) | 0.10 | 0.05 | 0.15 | Beta | Weissman *et al.* [8] |
| Suture needle, assorted sizes, round body | 0.75 | 0.38 | 1.00 | Beta | Weissman *et al.* [8] |
| Suture, catgut chromic 0, 150cm | 0.75 | 0.38 | 1.00 | Beta | Weissman *et al.* [8] |
| Suture, catgut chromic 1, 150cm | 0.75 | 0.38 | 1.00 | Beta | Weissman *et al.* [8] |
| Suture, catgut ob sterile w/needle | 0.75 | 0.38 | 1.00 | Beta | Weissman *et al.* [8] |
| Suture, catgut plain 2/0, 150cm | 0.75 | 0.38 | 1.00 | Beta | Weissman *et al.* [8] |
| Syringe, 5ml, dispos+needle+swab | 0.80 | 0.40 | 1.00 | Beta | Weissman *et al.* [8] |
| Syringe, 5ml, dispos+needle+swab | 0.20 | 0.10 | 0.30 | Beta | Weissman *et al.* [8] |
| Water for injection, 5ml | 0.20 | 0.10 | 0.30 | Beta | Weissman *et al.* [8] |
|  |  |  |  |  |  |
| ***Health resource use for normal delivery at health center*** |  |  |  |  |  |
| Average auxiliary health worker hours per patient | 2.00 | 1.00 | 3.00 | Exponential | Weissman *et al.* [8] |
| Average nurse/midwife hours per patient | 4.00 | 2.00 | 6.00 | Exponential | Weissman *et al.* [8] |
| Average length (in days) of hospital stay | 3 | 2 | 5 | Exponential | Weissman *et al.* [8] |
|  |  |  |  |  |  |
| *Proportion of patients using resource* |  |  |  |  |  |
| Lidocaine 2%, injection 50ml | 0.50 | 0.25 | 0.75 | Beta | Weissman *et al.* [8] |
| Paracetamol, tablets 500mg | 0.50 | 0.25 | 0.75 | Beta | Weissman *et al.* [8] |
| Tetracycline eye ointment | 1.00 | 0.50 | 1.00 | Beta | Weissman *et al.* [8] |
| Gauze pad, sterile, 12ply 76x76 | 0.50 | 0.25 | 0.75 | Beta | Weissman *et al.* [8] |
| Gloves, examination, latex, sterile, pair | 1.00 | 0.50 | 1.00 | Beta | Weissman *et al.* [8] |
| Mucus extractor | 0.05 | 0.03 | 0.08 | Beta | Weissman *et al.* [8] |
| Povidone iodine solution 10% 100mL | 1.00 | 1.00 | 1.00 | Beta | Weissman *et al.* [8] |
| Stationery - delivery record (clinic based) | 1.00 | 1.00 | 1.00 | Beta | Weissman *et al.* [8] |
| Suture, catgut ob sterile w/needle | 0.50 | 0.25 | 0.75 | Beta | Weissman *et al.* [8] |
| Syringe, 10ml, dispos+needle+swab | 0.50 | 0.25 | 0.75 | Beta | Weissman *et al.* [8] |
| Syringe, 5ml, dispos+needle+swab | 1.00 | 0.50 | 1.00 | Beta | Weissman *et al.* [8] |
| Umbilical tape | 1.00 | 0.50 | 1.00 | Beta | Weissman *et al.* [8] |
| Overnight stay | 0.50 | 0.25 | 0.75 | Beta | Weissman *et al.* [8] |
|  |  |  |  |  |  |
| ***Health resource use for normal delivery at hospital*** |  |  |  |  |  |
| Average auxiliary health worker hours per patient | 2.00 | 1.00 | 3.00 | Exponential | Weissman *et al.* [8] |
| Average nurse/midwife hours per patient | 4.00 | 2.00 | 6.00 | Exponential | Weissman *et al.* [8] |
| Average length (in days) of hospital stay | 3 | 2 | 5 | Exponential | Weissman *et al.* [8] |
|  |  |  |  |  |  |
| *Proportion of patients using resource* |  |  |  |  |  |
| Lidocaine 2%, injection 50ml | 0.50 | 0.25 | 0.75 | Beta | Weissman *et al.* [8] |
| Paracetamol, tablets 500mg | 0.50 | 0.25 | 0.75 | Beta | Weissman *et al.* [8] |
| Tetracycline, eye ointment 1% | 1.00 | 1.00 | 1.00 | Beta | Weissman *et al.* [8] |
| Gauze pad, sterile, 12ply 76x76 | 0.50 | 0.25 | 0.75 | Beta | Weissman *et al.* [8] |
| Gloves, examination, latex, sterile, pair | 1.00 | 1.00 | 1.00 | Beta | Weissman *et al.* [8] |
| Mucus extractor | 0.05 | 0.03 | 0.08 | Beta | Weissman *et al.* [8] |
| Povidone iodine solution 10% 100mL | 1.00 | 1.00 | 1.00 | Beta | Weissman *et al.* [8] |
| Stationery - delivery record (clinic based) | 1.00 | 1.00 | 1.00 | Beta | Weissman *et al.* [8] |
| Suture, catgut ob sterile w/needle | 0.50 | 0.25 | 0.75 | Beta | Weissman *et al.* [8] |
| Syringe, 10ml, dispos+needle+swab | 0.50 | 0.25 | 0.75 | Beta | Weissman *et al.* [8] |
| Syringe, 5ml, dispos+needle+swab | 1.00 | 1.00 | 1.00 | Beta | Weissman *et al.* [8] |
| Umbilical tape | 1.00 | 1.00 | 1.00 | Beta | Weissman *et al.* [8] |
| Overnight stay in hospital | 0.50 | 0.25 | 0.75 | Beta | Weissman *et al.* [8] |
|  |  |  |  |  |  |
| ***Health resource use for uterine rupture at hospital*** |  |  |  |  |  |
| *Average health worker hours per patient* |  |  |  |  |  |
| Laboratory assistant | 0.25 | 0.2 | 0.3 | Exponential | Weissman *et al.* [8] |
| Anaesthesist | 1 | 0.5 | 1.5 | Exponential | Weissman *et al.* [8] |
| Auxiliary/Attendant | 4.5 | 2.25 | 6.75 | Exponential | Weissman *et al.* [8] |
| Nurse/Midwife | 2 | 1.00 | 3.00 | Exponential | Weissman *et al.* [8] |
| Obstetrician | 1 | 0.50 | 1.50 | Exponential | Weissman *et al.* [8] |
| Average length (in days) of hospital stay | 14 | 7 | 21 | Exponential | Assumed |
|  |  |  |  |  |  |
| *Proportion of patients utilizing resource* |  |  |  |  |  |
| Blood, one unit | 20% | 16% | 24% | Beta | Weissman *et al.* [8] |
| Ampicillin, injection 1g | 100% | - | - | - | Weissman *et al.* [8] |
| Povidone iodine solution 10% 100mL | 100% | - | - | - | Weissman *et al.* [8] |
| Halothane gas | 100% | - | - | - | Weissman *et al.* [8] |
| Paracetamol, tablets 500mg | 100% | - | - | - | Weissman *et al.* [8] |
| Pethidine 50mg/ml, injection 1ml | 100% | - | - | - | Weissman *et al.* [8] |
| Sodium lactate (Ringer) + set, solution 500ml | 100% | - | - | - | Weissman *et al.* [8] |
| Suxamethonium 50mg/ml, injection 2ml | 100% | - | - | - | Weissman *et al.* [8] |
| Thiopental, injection 1g | 100% | - | - | - | Weissman *et al.* [8] |
| Blood giving set with needle, disposable | 100% | - | - | - | Weissman *et al.* [8] |
| Catheter, foley | 100% | - | - | - | Weissman *et al.* [8] |
| Gauze pad, sterile, 12ply 76x76 | 100% | - | - | - | Weissman *et al.* [8] |
| Gloves, surgeons, disposable, pair | 100% | - | - | - | Weissman *et al.* [8] |
| Infusion giving set with needle, disposable | 100% | - | - | - | Weissman *et al.* [8] |
| Oxygen, 100 liters | 100% | - | - | - | Weissman *et al.* [8] |
| Oxygen, 100 liters | 100% | - | - | - | Weissman *et al.* [8] |
| Suture, catgut ob sterile w/needle | 100% | - | - | - | Weissman *et al.* [8] |
| Suture, catgut plain 2/0, 150cm | 100% | - | - | - | Weissman *et al.* [8] |
| Syringe, 5ml, dispos+needle+swab | 100% | - | - | - | Weissman *et al.* [8] |
| Water for injection, 5ml | 100% | - | - | - | Weissman *et al.* [8] |
| Blood group test | 100% | - | - | - | Weissman *et al.* [8] |
| Haemoglobin test | 100% | - | - | - | Weissman *et al.* [8] |
| Overnight hospital stay | 100% | - | - | - | Weissman *et al.* [8] |
| NMS refers to National Medical Stores, MSH refers to Management Sciences for Health | | | | | |

References

1. National Medical Stores (2010) Price Indicator. Available from [http://www.NMS .go.ug/images/imported_files/CATALOG_OCT_2010.pdf](http://www.nms.go.ug/images/imported_files/CATALOG_OCT_2010.pdf) Accessed November 13 2013.

2. Adam T, Evans DB, Murray CJ (2003) Econometric estimation of country-specific hospital costs. Cost Eff Resour Alloc 1: 3.

3. Lara AM, Kandulu J, Chisuwo L, Kashoti A, Mundy C, et al. (2007) Laboratory costs of a hospital-based blood transfusion service in Malawi. J Clin Pathol 60: 1117-1120.

4. Ninci A, Ocakacon R (2004) How much do lab tests cost? Analysis of Lacor Hospital laboratory services. Health Policy and Development. pp. 114-150.

5. Matsiko CW, Kiwanuka J (2003) A review of Human Resources for Health in Uganda. Health Policy and Development 1: 15-20.

6. Management Sciences for Health (2014) International Drug Price Indicator Guide. Available from <http://erc.msh.org/dmpguide/pdf/DrugPriceGuide_2012_en.pdf> Accessed January 17 2014.

7. Levin A, Dmytraczenko T, McEuen M, Ssengooba F, Mangani R, et al. (2003) Costs of maternal health care services in three anglophone African countries. Int J Health Plann Manage 18: 3-22.

8. Weissman E, Sentumbwe-Mugisa O, Mbonye AK, Kayaga E, Kihuguru SM, et al. (1999) Uganda Safe Motherhood Programme Costing Study. Geneva: World Health Organization and Ministry of Health, Uganda. Available from <http://whqlibdoc.who.int/hq/1999/WHO_CHS_RHR_99.9.pdf?ua=1> Accessed November 12, 2013.
