# Supplementary material for: Potential Cost-Effectiveness of Prenatal Distribution of Misoprostol for Prevention of Postpartum Hemorrhage in Uganda
Source: PLoS One. 2015 Nov 11;10(11):e0142550. doi: 10.1371/journal.pone.0142550 (PMC4641649; doi:10.1371/journal.pone.0142550)
Supplement: S2 Table — Vaginal delivery costs are calculated according to delivery pathway trajectory (DOCX) [file pone.0142550.s002.docx]

S2 Table. Summary of cost estimates for the outcomes—vaginal delivery, postpartum hemorrhage, uterine rupture and stillbirth—in the model. Vaginal delivery costs are calculated according to delivery pathway trajectory

|  | ***Vaginal Delivery*** | | | | | ***PPH*** | ***Uterine Rupture*** | ***Still birth*** |
| --- | --- | --- | --- | --- | --- | --- | --- | --- |
| ***Item*** | ***HC*** | ***Hospital*** | ***TBA*** | ***Friend/Relative*** | ***Unassisted*** |  |  |  |
| *Direct medical costs* |  |  | 11.77 |  |  |  |  |  |
| Drugs & sundries | 3.77 | 3.77 | - | - | - | 26.71 | 52.73 | 3.77 |
| Health worker time | 5.95 | 5.95 | - | - | - | 11.16 | 12.52 | 5.95 |
| Capital & overheads | 5.51 | 5.75 | - | - | - | 26.83 | 53.66 | 5.75 |
| *Direct non-medical costs* | 4.16 | 11.91 | 1.39 | - | - | 11.91 | 11.91 | 11.91 |
| *Indirect (time) costs* | 6.41 | 6.41 | 6.22 | 6.22 | 6.41 | 14.70 | 29.20 | 6.41 |
| ***Total cost*** |  |  |  |  |  |  |  |  |
| *Government perspective* | 15.23 | 15.47 | - | - | - | 64.70 | 118.91 | 15.47 |
| *Societal perspective* | 25.81 | 33.80 | 19.37 | 6.22 | 6.41 | 91.31 | 160.02 | 33.80 |
