# Supplementary material for: Potential Cost-Effectiveness of Prenatal Distribution of Misoprostol for Prevention of Postpartum Hemorrhage in Uganda
Source: PLoS One. 2015 Nov 11;10(11):e0142550. doi: 10.1371/journal.pone.0142550 (PMC4641649; doi:10.1371/journal.pone.0142550)
Supplement: S3 Table — (DOCX) [file pone.0142550.s003.docx]

S3 Table. Misoprostol program costing model

| ***Item*** | ***Quantity*** | ***Rate*** | ***Total*** |
| --- | --- | --- | --- |
| Misoprostol 600mcg dose | 10,000 | $ 0.3576 | $ 3,576 |
| Health worker training | 200 for 5 days | $ 10 per day | $ 10,000 |
| Training of mothers | 20 Nurse Full Time Equivalents | $ 2150 | $ 42,989 |
| Additional packaging cost | 10,000 | 50% x $ 0.3576 | $ 1,788 |
| ***Total cost (for 10,000 mothers)*** |  |  | ***$58,353*** |
| ***Mean cost (for each mother)*** |  |  | ***$5.835*** |
